# The Association between Resistance and Virulence of *Klebsiella pneumoniae* in High-Risk Clonal Lineages ST86 and ST101

**DOI:** 10.3390/microorganisms12101997

**Published:** 2024-09-30

**Authors:** Irina Pristas, Josip Ujevic, Kristian Bodulić, Natasa Andrijasevic, Branka Bedenic, Marina Payerl-Pal, Edita Susic, Karolina Dobrovic, Sien De Koster, Surbhi Malhotra-Kumar, Arjana Tambic Andrasevic

**Affiliations:** 1 University Hospital for Infectious Diseases, 10000 Zagreb, Croatia; josip.ujevic@gmail.com (J.U.); kristian.bodulic@gmail.com (K.B.); natasa.andrijasevic@gmail.com (N.A.); atambic@bfm.hr (A.T.A.); 2 Dental School of Medicine, 10000 Zagreb, Croatia; 3 Medical Microbiology Department, School of Medicine, University of Zagreb, 10000 Zagreb, Croatia; branka.bedenic@kbc-zagreb.hr; 4 BIMIS-Biomedical Research Center Šalata, School of Medicine, University of Zagreb, 10000 Zagreb, Croatia; 5 Department of Clinical and Molecular Microbiology, University Hospital Centre Zagreb, 10000 Zagreb, Croatia; 6 Public Health Institute of Medimurje County, 40000 Cakovec, Croatia; marina.payerl-pal@zzjz-ck.hr; 7 Public Health Institute of Šibenik and Knin County, 22000 Šibenik, Croatia; edita.susic@zzjz-sibenik.hr; 8 Clinical Hospital Dubrava, 10000 Zagreb, Croatia; kdobrov@kbd.hr; 9 Laboratory of Medical Microbiology, Vaccine and Infectious Disease Institute, University of Antwerp, 2000 Antwerp, Belgium; sien.dekoster@uza.be (S.D.K.); surbhi.malhotra@uantwerpen.be (S.M.-K.)

**Keywords:** *Klebsiella pneumoniae*, hypervirulence, virulence factors, resistance factors

## Abstract

*Klebsiella pneumoniae* is an opportunistic pathogen known for two main pathotypes: classical *K. pneumoniae* (cKp), often multidrug-resistant and common in hospitals, and hypervirulent *K. pneumoniae* (hvKp), associated with severe community-acquired infections. The recent emergence of strains combining hypervirulence and resistance is alarming. This study investigates the distribution of sequence types (STs), resistance, and virulence factors in *K. pneumoniae* strains causing bloodstream and urinary tract infections in Croatia. In 2022, 200 consecutive *K. pneumoniae* isolates were collected from blood and urine samples across several Croatian hospitals. Whole genome sequencing was performed on 194 isolates. Within the analyzed *K. pneumoniae* population, the distribution of sequence types was determined with multi-locus sequence typing (MLST) and capsule loci, resistance, and virulence determinants were assessed with the bioinformatics tool Kleborate. The analysis identified 77 different STs, with ST101 (24.6%) being the most prevalent, predominantly linked to the K17 capsular type (CT), invasive device usage, high antimicrobial resistance, and low virulence scores. The highest virulence scores were recorded in ST86 isolates, which were predominantly linked to the K2 CT and included some strains with medium resistance scores. String tests were positive in 19 strains, but only four of those harbored hypermucoviscous genetic determinants. The most prevalent ST101 clone in Croatia demonstrated a diverging association between resistance and virulence. An alarming co-existence of resistance and virulence was recorded in the ST86 strains.

## 1. Introduction

*Klebsiella pneumoniae* (*K. pneumoniae*) is a widely recognized opportunistic pathogen frequently responsible for healthcare-associated infections in critically ill patients, immunocompromised individuals, and patients with compromised mucocutaneous barriers. This pathogen can cause urinary and respiratory tract, bloodstream, and central nervous system infections. The severity of *K. pneumoniae* infections can be influenced by several factors, including the patient’s overall health, underlying conditions, as well as virulence and antimicrobial resistance profiles of the infecting strain [1,2,3].

*K. pneumoniae* exhibits the capacity of acquiring new genetic material, resulting in the evolution and circulation of two specific pathotypes of *K. pneumoniae*. These pathotypes include the above mentioned classical pathotype (cKp), mostly causing healthcare-associated infections in Europe and America, and the hypervirulent pathotype (hvKp), described in Asian and Pacific countries as the cause of severe and often fatal community-acquired infections [1,4,5]. The first case of a hvKp strain causing severe infection in a previously healthy individual was described in 1986 in Taiwan. This patient presented as a liver abscess with metastatic spread to the eye, causing endophthalmitis [6]. Since then, hvKp has been increasingly recognized as a pathogen capable of causing severe community-acquired infections in healthy individuals, such as sepsis with metastatic lesions, liver abscess, endophthalmitis, and meningitis [7,8,9,10,11]. HvKp is the leading cause of liver abscesses in China, South Korea, Taiwan, and Singapore, and up to 40% of stated infections have been associated with nosocomial spread [12,13]. Concerningly, hvKp infections have started to spread to countries beyond the initially described regions, with both pathotypes being increasingly present globally [1,11,13,14,15,16].

In the era of the global antibiotic resistance problem, cKp has been recognized as a significant pathogen due to its ability of acquiring resistance genes and rapidly spreading in the hospital setting [17,18]. Of special concern is the resistance to carbapenems and carbapenem-resistant *K. pneumoniae* (CRKP), which is becoming one of the most common healthcare-associated difficult-to-treat pathogens [19,20,21,22]. The most notable mechanism of carbapenem resistance is the production of carbapenemases, such as *Klebsiella pneumoniae* carbapenemase (KPC), metallo-β-lactamases (MBL) belonging to the IMP, VIM, and NDM family, and carbapenem-hydrolyzing oxacillinases (CHDL), often associated with porin loss or upregulation of efflux pumps.

High levels of resistance to carbapenems have often been attributed to the global spread of a single clonal group (CG), such as CG258, which includes sequence types (STs) 258, 512, and 11 [23,24,25]. However, more recent studies suggest the spread of other emerging highly resistant clones, including ST101, 147, 307, 231, and 383 [24,25].

Previously, hvKp strains exhibited low antibiotic resistance rates and were suitable for treatment with numerous antibiotics [1,13,14]. Recently, resistance genes were described in hypervirulent strains, contributing to the hospital outbreaks caused by hvKp [26,27]. The most common carbapenemase genes described were *bla*_KPC_, *bla*_OXA-48_, *bla*_NDM_, *bla*_VIM_, and *bla*_IMP_. Several hospital epidemics caused by carbapenemase-producing hypervirulent *K. pneumoniae* strains have already been reported in China [1,4,9,10,12,26,27]. Although carbapenem-resistant hypervirulent *K. pneumoniae* (CR-hvKp) is more common in Asian countries, its global spread has been recorded since 2010 [1,8,14,15,19,20,21,25,28,29,30,31]. Hypervirulent *K. pneumoniae* strains are usually associated with certain clonal lineages, such as ST23, ST65, and ST86, with recent reports raising concern of the global spreading of CR-hvKP ST23 [1,8,14,15,19,20,21,25,28,30,31].

The emergence of CR-hvKP highlights the urgent need for epidemiological and clinical studies describing the genetic background of circulating *K. pneumoniae* strains. Taking into consideration that hvKp and CR-hvKp strains have already been detected in Europe [1,8,14], we aimed to perform an explorative molecular epidemiological study on *K.pneumoniae* strains causing bloodstream infections (BSI) and urinary tract infections (UTI) in Croatia.

## 2. Materials and Methods

### 2.1. Clinical Sampling

Throughout 2022, 200 *K. pneumoniae* isolates were collected from blood cultures and urine. Strains were collected from different regions in Croatia: University Hospital for Infectious Diseases “Dr. Fran Mihaljevic”, Clinical Hospital Merkur, and Clinical Hospital Dubrava, in Zagreb, the Public Health Institute of Medimurje County, and the Public Health Institute of Sibenik-Knin County.

The isolates were obtained in adherence to the clinical management protocols and routine indications for collecting such samples. Specifically, blood cultures were collected from patients suspected of having sepsis, and urine samples were obtained from patients suspected of suffering from UTI. Alongside each isolate, relevant information such as type of specimen, hospital, and the sampling date were collected. Moreover, the presence of central venous catheter, urinary catheter, and mechanical ventilation utilization were documented, providing indirect information on probable healthcare-associated infections.

### 2.2. Isolate Processing

Clinical samples were cultured according to good laboratory practice. Individual colonies were selected for identification using matrix-assisted laser desorption-ionization time-of-flight mass spectrometry (MALDI-TOF, Bruker Diagnostics, Bremen, Germany). The string test was performed to identify *K. pneumoniae* strains exhibiting the hypermucoviscous (hmv) phenotype. The test was performed using a sterile loop to lift the colony from the surface. If a viscous string longer than 5 mm was formed when the loop was lifted, the strain was considered to be hmv [1].

### 2.3. DNA Extraction, Library Construction and Sequencing

Total genomic DNA was extracted from the selected colonies that had been cultured overnight at 37 °C on blood agar plates. DNA extraction was performed using the MasterPureTM Complete DNA and RNA Purification Kit (Epicenter, Madison, WI, USA), following the manufacturer’s instructions. Additionally, DNA was purified using the DNA Clean and Concentrator TM-10 Kit (Zymo Research, Tustin, CA, USA). Genomic libraries were prepared with the Nextera XT DNA Library Preparation Kit (Illumina, Inc., San Diego, CA, USA). The Illumina MiSeq platform (Illumina Inc., San Diego, CA, USA) was used for the 2 × 250 bp paired-end sequencing.

### 2.4. Genotypic Characterization of K. pneumoniae Isolates

Quality of short reads was assessed using FastQC (v0.12.1) [32]. CheckM (v1) was employed to estimate the completeness of the analyzed genomes and possible contamination (Parks DH). For the analysis of the raw sequencing data, we utilized BacPipe (v2.6.1) [33]. Briefly, sequencing data was trimmed with Trim Galore (https://github.com/FelixKrueger/TrimGalore, accessed on 25 August 2024), followed by de novo assembly of the reads with SPAdes (v3.13) [34]. The assembled genomic data was annotated using Prokka (v1.14.6) [35]. To genetically profile the isolates, multi-locus sequence typing (MLST) analysis was conducted, determining the sequence type (ST) by aligning nucleotides with the established seven-locus *K. pneumoniae* scheme using BLAST (v2.4.17) [36]. Kleborate (v2.3.2) was utilized to detect the presence of resistance and virulence determinants, as well as for capsule typing and species identification [37]. This tool assigns score values ranging from 1 to 3 for loci associated with clinically significant antibiotic resistance progressing from ESBL to carbapenemase, and then to carbapenemase combined with colistin resistance, and from 1 to 5 for those linked to virulence determinants (0 = no yersinabactin, colibactin, or aerobactin; 1 = yersiniabactin only; 2 = yersiniabactin and colibactin (or colibactin only); 3 = aerobactin without yersiniabactin or colibactin; 4 = aerobactin with yersiniabactin (no colibactin); 5 = yersiniabactin, colibactin, and aerobactin. Lastly, using the chewBBACA (v3.3.10) software, cgMLST analysis was employed to determine the genetic relatedness and population structure of *K. pneumoniae* strains, enabling a comprehensive understanding of strain evolution and epidemiological insights [38]. To perform chewBBACCA’s Allele Calling algorithm, an external scheme of *K. pneumoniae* (cgMLST scheme can be found at https://www.cgmlst.org/ncs/schema/Kpneumoniae3259, accessed on 25 August 2024) was imported from cgMLST. The total number of loci used in this scheme was 2358. Generated distance matrix was used to construct a neighbor-joining tree (NJ) with Grapetree software (v1.5.0) [39]. Visualization of the cgMLST results was performed using the online platform iTOL (https://itol.embl.de/, accessed on 26 August 2024) [40].

### 2.5. Statistical Analysis

Numerical variables with non-normal distributions were reported using medians and ranges, while categorical variables were reported using numbers and percentages. Non-normal numerical variables were compared using the Mann–Whitney U test in case of two-group comparisons and with the Kruskal–Wallis test in case of multiple-group comparisons. The association of categorical variables was assessed using the chi-square test or Fisher’s exact test, as appropriate. All tests were two-tailed with the significance level set to 95% (*p* < 0.05). Statistical analysis was performed using R (version 4.1.2; R Core Team (2021)) and the ggplot2 package (version 3.4.2) [41,42].

## 3. Results

For this study, a total of 200 bacterial isolates from blood and urine were collected and identified as *K. pneumoniae*. Genomic data were obtained from 194 (97.0%) collected isolates, and in silico species identification confirmed the previously reported species identification of *K. pneumoniae sensu stricto* for 183 (94.3%) of these isolates, which were included in further analysis. The rest of the isolates, identified in silico as *K. quasipneumoniae*, or *K. variicola,* were excluded from the study. ST profiling identified a total of 77 different ST types, the majority of which were represented by a single isolate (70%). Within the dataset, a relatively high level of diversity was demonstrated (Simpson Diversity Index = 0.926). The most prevalent clonal lineage belonged to ST101 and was detected in 45 (24.6%) isolates. This was followed by ST37 and ST437 lineages, which were both detected in 8 (4.4%) isolates. In general, no ST grouping per site was noted (*p* > 0.05), as most of the lineages were unique across the analyzed institutions. Further analysis was based on the cgMLST-generated distance matrix. Out of the 2358 loci initially provided by the imported scheme for *K. pneumoniae*, 1626 loci were successfully assigned to all analyzed isolates. According to the cgMLST95 scheme, only loci assigned to 95% or more of the analyzed isolate population (2123 loci) were considered for further analysis.

**Figure 1:**
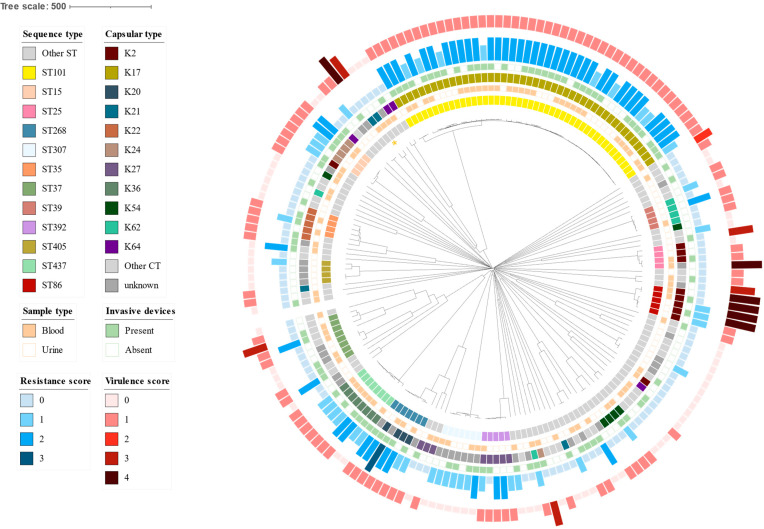
Neighbor-Joining tree based on a cgMLST distance matrix of 183 *K. pneumoniae* genomes, with annotations as shown in the figure legend. Resistance and virulence scores are represented by inner blue and outer red bar plots, respectively, with size and color indicating score variations. Sequence types are shown in the inner-colored strip, while the outer color strip displays capsular types. The presence of invasive devices and sample types are denoted by a binary box data frame. Yellow asterisk marks the ST290 strain, detected in two isolates.

In terms of the antimicrobial resistance score, most isolates (48.6%) were classified with a resistance score of 0 (51.5%), followed by a score of 2 (28.4%), a score of 1 (22.4%), and a score of 3 (0.56%). Regarding the virulence scores, the majority of isolates displayed a score of 1 (59.0%), followed by isolates with scores of 0 (34.4%), 4 (3.0%), 3 (2.7%), 2 (0.5%), and 5 (0%). The association between the most common STs and sample type, presence and absence of invasive devices, virulence scores and antimicrobial resistance scores are presented in Figure 1 and Table 1. The most prevalent clonal lineage, ST101, was associated with the presence of invasive devices (*p* < 0.001). Accordingly, isolates from patients requiring invasive devices were significantly more likely to belong to the ST101 sequence type (OR = 3.7, 95% CI 1.7–8.2, *p* < 0.001). We also found a significant association between ST101 and virulence score (*p* < 0.001), with all ST101 isolates (45, 100.0%) exhibiting a virulence score of 1. Furthermore, the resistance scores of ST101 isolates were higher (resistance scores of 1 or higher (*p* < 0.001)), with all resistant isolates associated with the presence of exclusively ESBL (9/45, 20.0%) only (*bla*_CTX-M-15_) or carbapenemase (36/45, 80.0%) genes (*bla*_OXA-48_, *bla*_OXA-244_, *bla*_KPC-2_). Furthermore, ST101 isolates were significantly more likely to produce carbapenemases than other isolates (OR = 27.6 95% CI 10.9–77.6, *p* < 0.001). The most prevalent clonal lineage associated with higher virulence scores was ST86 (5, 2.7%). We did not find a significant association between the ST86 isolates and invasive device utilization (*p* = 0.677). Four (80.0%) of ST86 isolates exhibited high virulence scores of 4, while one isolate displayed a virulence score of 1. As such, ST86 isolates were significantly more likely to exhibit a virulence score of 4 compared to other isolates (OR = 265, 95% CI 18.3–14,644.4, *p* < 0.001). When considering all isolates, 6 (3.3%) isolates were associated with a virulence score of 4 and were distributed among specific sequence types as follows: ST86 (4/183, 2.2%), ST25 (1/183, 0.5%) and ST290 (1/183, 0.5%). On the contrary, all ST86 isolates exhibited low antibiotic resistance scores (0 or 1). While carbapenem resistance was not observed in ST86 strains, a relatively high percentage of ST86 isolates (3/5, 60%) harbored ESBL genes (*bla*_CTX-M-15_) responsible for the resistance to a broad spectrum of β-lactam antibiotics.

Figure 1 also illustrates the distribution of predicted capsular serotypes (CT) and STs. The capsular serotypes of the analyzed isolates were highly diverse, consisting of 37 different K-loci for 147/183 (80.3%) of the analyzed isolates. The remaining 36/189 (19.7%) of the isolates were designated as having an unknown K-type. The most prevalent K-type was K17, linked to ST101 and identified in 45 isolates. The next most common K-type was K2, associated with multiple STs (ST86, ST25, ST34) and detected in 10 isolates. Most STs were linked to a single K-locus with the exceptions of ST461, ST20, and ST37, which included several K-types each. K2 isolates were significantly more likely to be associated with higher virulence scores compared to other K-types (OR = 126.00, 95% CI 11.90–6404.90, *p* < 0.001).

Table 2 and Table 3 present the direct association between antimicrobial resistance and virulence scores and the relation of those scores to clinical parameters. In general, specimen type was not associated with any of the analyzed parameters, with the exception of isolates with a virulence score of 0 being more frequently isolated in urine than in blood (*p* = 0.004). Invasive device usage was significantly more common in patients infected with isolates resistant to carbapenemases (resistance score 2, *p* < 0.001) and in patients with a virulence score of 1 (*p* < 0.001). We also observed a significant negative correlation between resistance and virulence scores. Isolates with higher virulence scores (3–5) more commonly exhibited a resistance score of 0 (*p* = 0.029). Finally, hypervirulent isolates with a virulence score of 4 were significantly more likely to exhibit a positive string test result (*p* = 0.001).

**Figure 2:**
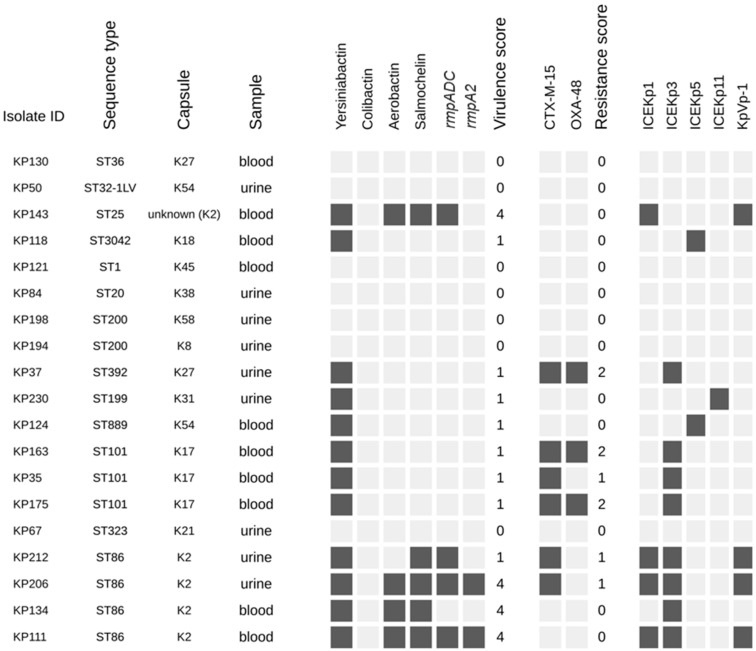
The analysis of the 19 *K. pneumoniae* isolates that tested positive for the string test. Black squares indicate the presence of virulence and resistance determinants.

Hmv isolates of *K. pneumoniae* identified using the string test method were further analyzed. Nineteen string-test positive samples were screened for virulence factors and resistance determinants. As shown in Figure 2, these isolates exhibited a diverse profile of STs and CTs. No correlation was observed between sample of origin and the hmv phenotype (*p* = 0.970). The association between virulence factors and the string test results is shown in Table 4. Positive string test results were significantly more common in isolates harboring *iuc* (*p* = 0.016), *iro* (*p* < 0.001), *rmpADC* (*p* = 0.002) and *rmpA2* (*p* = 0.029). As such, yersiniabactin was the most prevalent virulence factor, detected in 12/19 (63.1%) of the hmv isolates. Other factors such as aerobactin (4/19, 21.0%), salmochelin (5/19, 26.3%), *rmpADC* (4/19, 21.0%), and *rmpA2* (2/19,10.5%) were associated with ST86 and ST25, with both clonal lineages belonging to K2 type. In total, seven (36.8%) hmv isolates contained the *rmpA* gene, a regulator of the mucoid phenotype. Four (57.4%) out of those isolates were string-test positive. Furthermore, four (57.4%) *rmpA*-positive isolates belonged to ST86, two (28.6%) isolates were identified as ST25, and one (14.3%) isolate belonged to ST412. Almost all (6, 85.7%) of the *rmpA*-positive isolates exhibited the K2 serotype. Notably, four (57.4%) hmv isolates had a virulence score of 4.

## 4. Discussion

Findings presented in this study reveal significant insights into the molecular epidemiology of clinical *K. pneumoniae* strains in Croatia, with a particular focus on high-risk clonal lineages ST86 and ST101. This study offers valuable insights into the distribution of sequence types, resistance, and virulence profiles within the analyzed *K. pneumoniae* population.

Our data demonstrate a high level of ST diversity among clinical *K. pneumoniae* isolates, reflecting the genetic variability of this pathogen. The predominance of ST101, comprising 24.6% of our isolates, aligns with global reports highlighting the emergence and spread of this high-risk clone [16,20,25,28,29,43,44]. According to the literature, as well as our analysis, many studies identified the major role of ST101 in the dissemination of CRKP isolates across Europe and other regions [20,25,45]. Infections caused by CRKP have been associated with higher mortality and morbidity rates, prolonged hospitalization, and increased treatment costs [19]. Indeed, in our dataset, ST101 isolates were significantly more likely to produce carbapenemases than other isolates. Among the analyzed ST101 isolates, the predominant carbapenemase was OXA-48, which is consistent with the studies coming from Balkan region [45,46]. However, in several other studies, KPC was the predominant carbapenemase [20]. Our study shows that ST101 is the most common strain found in bloodstream infections (BSIs) and is closely linked to the hospital environment, particularly where invasive devices are used. This emphasizes the significant clinical challenges presented by this strain, particularly in the hospital environment. The correlation between ST101 and invasive infections has been documented in previous studies, further emphasizing the need for proper infection control measures to curb the spread of this high-risk clone [19,20,21,22,23]. In our study, ST437 was the only clone more likely to be associated with BSI than UTIs. Interestingly, all of the ST101 isolates displayed low virulence scores, implying that such strains, although predominant in this study, still do not harbor virulence factors. This is in contrast to recently published findings coming from other parts of Europe [14,16,20,25,28,29,43,44,45,47]. This study indicates that the high rate of ST101 infections is attributable to multidrug-resistant phenotypes, as well as lapses in infection control and manipulation with invasive devices, rather than to the high virulence of this clone.

Various carbapenemase genes have been detected in hvKp isolates in recent years, including *bla*_OXA-48-like_, *bla*_KPC_, *bla*_NDM_, and *bla*_VIM_ [14]. The hvKp ST23-K1 isolates from the EU/EEA mainly acquired *bla*_OXA-48-like_ carbapenemase genes. In addition, hvKp ST23 isolates resistant to last-resort antibiotics such as colistin or ceftazidime-avibactam have been recorded [37,45,48]. Colistin-resistant ST101 strains have been described recently and associated with high mortality rates, where the ST101 type was found to be a significant independent predictor of patient mortality [45,46]. Notably, in our study, only one strain was colistin-resistant. The strain belonged to the ST268 clone. The resistance was found to be due to the partial truncation of the *pmrB* gene, and the strain harbored the *bla*_OXA-48_ gene as well, giving it a resistance score of 3. The virulence score of that strain was 1. In a recently published report from southern Croatia, the emergence and clonal spread of a high-risk ST101/KPC-2 clone of *K. pneumoniae* resistant to colistin was highlighted, demonstrating that colistin-resistant ST101 is already present in Croatia [49].

Consistent with the diversity of STs, the capsular serotypes were also highly diverse. The most prevalent K-type was K17, linked to ST101, which is described in the similar studies [25,47]. Also, similar to findings in other studies, most STs were linked to a single K-locus [25,44,50].

The second most common K-type in our dataset was K2, associated with multiple STs, and in particular, STs exhibiting high virulence scores (ST86, ST25, ST34). Association of K2 with ST86, is concordant with similar studies [31,44,51,52,53].

Despite the presence of hypervirulent *K. pneumoniae* for almost four decades, the definition of hypervirulence is not fully defined, largely due to the complexity of virulence mechanisms. So far, several virulence factors and different phenotypes that can serve as markers of hypervirulence have been described [1,4,7,8,14,31,37,44]. Many of these factors are contributing to the definition of a hypervirulence-hmv phenotype, presence of virulence genes, STs, capsule types (CTs), and clinical presentation [1,8]. However, there is a growing need for defining uniform hypervirulence criteria [48].

Several studies demonstrated that the hmv phenotype, originally detected by a string test, does not necessarily translate to a hypervirulent pathotype [54,55]. Moreover, studies suggest that the capsule type contributes to hypervirulence only to a certain extent [1]. The most common capsule types associated with hvKp are K1, K2, K5, and K57 [1,8,37,55]. In our study, the CT linked to the highest virulence score was K2 and it largely corresponded to virulent strains of ST86 and ST25 clonal lineages.

Historically, the string test was used predominantly for defining the hmv phenotype, and thus, the terms hypervirulent and hmv were often used as synonyms. Regulators of mucoid phenotype *rmpA/rmpA2*, together with a *magA* (mucoviscosity-associated gene), represent some of the frequently encountered virulence factors in hvKp strains. More than 50% of hvKp strains express either *rmpA* or *rmpA2*, as opposed to cKp (only 7–20%), potentially justifying the usage of *rmpA* as a virulence marker [1,8,14,44,48,54,55]. Our results demonstrated a significant number of string-test positive strains. However, not all of these strains were hypervirulent, which is consistent with the findings of the recent studies [1,8,48,54]. We confirmed that the majority of *rmpADC* and/or *rmpA2*-positive strains were mostly associated with higher virulence scores, certain STs (mostly ST86 and ST25), the K2 capsule type, and a positive string test result. The minority of *rmpADC* and/or *rmpA2*-positive strains have not shown hmv phenotype but displayed a virulence score of 4. Such findings were described in the more recent research where hmv phenotypes and increased capsule synthesis are shown to be independent phenomena [8,44,48,55].

Siderophores are molecules that are important for bacterial iron acquisition from the environment, and their presence is used as a virulence marker. In *K. pneumoniae*, representative siderophores include aerobactin (encoded by *iuc* genes), salmochelin (encoded by *iro* genes), and yersiniabactin (encoded by *ybt* genes) [1,8,14,37,44,48,55,56]. Among them, aerobactin is a commonly used marker for hypervirulence, although the use of a virulence scoring system proposed by Lam et al. has become a well-accepted method for defining hypervirulent genomes [8,15,55,56,57]. In our study, we graded the virulence of our *K. pneumoniae* strains using the Kleborate tool (v2.3.2) [37]. As such, we demonstrated that carbapenemase production was associated with isolates of low virulence scores.

In this study, ST101 isolates harbored different resistance genes and only the yersiniabactin (*ybt*) virulence gene. This gene is described to be present in both hvKp and cKp, and as a single virulence factor attributes to a low virulence score of 1. On the other hand, ST86 lineage exhibited a distinct virulence profile. Although not so numerous in our dataset, ST86 isolates were significantly more likely to exhibit higher virulence scores, predominantly a score of 4. Importantly, these isolates did not exhibit carbapenem resistance. These results mirror the findings from other regions, where ST86 was shown to be associated with hypervirulent phenotypes, often linked to severe community-acquired infections but originally susceptible to antibiotics [1,4,6,8,31,37]. However, in our data set, ST86 isolates were similarly found in patients with and without invasive devices, and some isolates demonstrated the presence of ESBL genes, suggesting that the virulent ST86 clone is not exclusively related to community-acquired infections. The wide usage of broad-spectrum cephalosporins might fuel the spread of this clone in hospital settings and in the community.

The global reports of CR-hvKp outbreaks, particularly in China, highlight the potential for these dual-threat strains to emerge and spread [58]. The spread of carbapenem-resistant hvKp in healthcare settings is expected to result in increased morbidity and mortality among patients who are already severely ill. Defining and detecting hvKp is vital for effective treatment and infection control. Such strains could pose significant therapeutic challenges, necessitating urgent development of new antimicrobial agents, new treatment options and vaccines, as well as enhanced surveillance strategies [14,55].

In conclusion, our study revealed a divergent relationship between resistance and virulence in the high-risk resistant *K. pneumoniae* ST101 clone, while the high-risk virulent ST86 clonal lineage did not exhibit such a clear-cut association between virulence and resistance. Although currently rare in Croatian hospitals, the co-existence of resistance and virulence determinants in some isolates is concerning and calls for continued and concerted molecular surveillance in our region.

## Figures and Tables

**Table 1:** The correlation between ST and different *K. pneumoniae* isolate parameters.

ST (*n* > 3)	Total	Blood	Urine	*p*-Value	I.d. Yes	I.d. No	*p*-Value	VS 0	VS 1	VS 2	VS 3–5	*p*-Value	RS 0	RS 1	RS 2–3	*p*-Value
ST101	45	28	17	0.060	33	12	<0.001	0	45	0	0	<0.001	0	9	36	<0.001
ST37	8	5	3	0.496	4	4	0.999	3	4	0	1	0.485	7	0	1	0.099
ST437	8	7	1	0.034	6	2	0.279	2	6	0	0	0.834	0	6	2	<0.001
ST268	7	3	4	0.999	5	2	0.682	0	7	0	0	0.150	0	2	5	0.005
ST307	7	4	3	0.999	4	3	0.999	7	0	0	0	0.002	0	6	1	<0.001
ST392	5	1	4	0.368	2	3	0.677	0	5	0	0	0.293	1	2	2	0.429
ST86	5	2	3	0.999	2	3	0.677	1	0	0	4	<0.001	2	3	0	0.107
ST15	4	2	2	0.999	3	1	0.621	0	4	0	0	0.4662	0	2	2	0.088
ST25	4	2	2	0.999	1	3	0.360	1	2	0	1	0.253	4	0	0	0.188
ST35	4	2	2	0.999	2	2	0.999	1	3	0	0	0.999	3	1	0	0.448
ST39	4	1	3	0.620	0	4	0.055	1	3	0	0	0.999	3	0	1	0.811
ST405	4	0	4	0.120	0	4	0.055	4	0	0	0	0.063	3	1	0	0.448
Other ST	78	34	44		32	46		43	29	1	5		66	9	3	

Abbreviations: ST, sequence type; *n*, number; I.d., invasive device; VS, virulence score; RS, resistance score.

**Table 2:** The correlation between antimicrobial resistance scores, specimen type and presence of invasive devices.

Antimicrobial Resistance Score	Blood	Urine	*p*-Value	Invasive Devices Yes	Invasive Devices No	*p*-Value	Virulence Score (0–2)	Virulence Score (3–5)	*p*-Value
0: none	39	50	0.139	32	57	<0.001	80	9	0.029
1: ESBL	20	21	0.999	24	17	0.375	39	2	0.999
2: carbapenemase	31	21	0.103	37	15	<0.001	52	0	0.035
3: carbapenemase + colistin	1	0	0.497	1	0	0.999	1	0	0.999

Abbreviations: ESBL, extended-spectrum beta-lactamases

**Table 3:** The correlation between virulence scores and specimen type and presence of invasive devices.

Virulence Score	Blood	Urine	*p*-Value	Invasive Devices Yes	Invasive Devices No	*p*-Value	Positive String Test	Negative String Test	*p*-Value
0: none	22	41	0.004	22	41	0.001	7	56	0.803
1: *ybt*	61	47	0.035	67	41	<0.001	8	100	0.140
2: *ybt* + *clb*	0	1	0.999	0	1	0.999	0	1	0.999
3: *iuc*	4	1	0.211	3	2	0.999	0	5	0.999
4: *ybt* + *iuc*	4	2	0.444	2	4	0.434	4	2	0.001
5: *ybt* + *clb* + *iuc*	0	0		0	0		0	0	

Abbreviations: *ybt*, yersiniabactin; *clb*, colibactin; *iuc*, aerobactin.

**Table 4:** The association between virulence factors and string test results.

Virulence Factor	String Test Positive Isolates 19 (10.4%)	String Test Negative Isolates 164 (89.6%)	OR (95% CI)	*p*-Value
none	7	56	1.12 (0.35–3.30)	0.803
*ybt*	12	103	1.01 (0.34–3.21)	0.999
*iuc*	4	7	5.87 (1.13–26.50)	0.016
*iro*	5	3	18.47 (3.22–131.16)	<0.001
*rmpADC*	4	3	13.86 (2.13–103.87)	0.002
*rmpA2*	2	1	18.39 (0.91–1121.10)	0.029

Abbreviations: *ybt*, yersiniabactin; *iuc*, aerobactin; *iro*, salmochelin; *rmpADC/A2*, regulator of mucoid phenotype.

## Data Availability

The data presented in this study are available on request from the corresponding author because the data are part of an ongoing research.
